# Shared Decision‐Making on Drinking Alcohol in Older Adults Living in Residential Care Facilities: Care Professionals' Perspectives

**DOI:** 10.1111/opn.70080

**Published:** 2026-04-20

**Authors:** Lisette de Graaf, Tineke Roelofs, Meriam Janssen, Sascha Bolt, Katrien Luijkx

**Affiliations:** ^1^ Department of Tranzo, School of Social and Behavioral Sciences Tilburg University Tilburg the Netherlands; ^2^ Mijzo Waalwijk the Netherlands; ^3^ Archipel Zorggroep Eindhoven the Netherlands

**Keywords:** nursing home, person‐centred care (PCC), substance use

## Abstract

**Introduction:**

Older adults living in residential care facilities (RCFs) may wish to drink alcohol, despite adverse outcomes. Shared decision‐making (SDM) could support care professionals in balancing residents' autonomy and well‐being with the adverse outcomes to all residents and staff. This study aims to assess factors that could affect shared decision‐making (SDM) and care professionals' behaviour regarding residents' alcohol use in residential care facilities (RCFs).

**Methods:**

A quantitative cross‐sectional study and explorative analyses were chosen to answer the research question. Care professionals working in psychogeriatric or in somatic units of RCFs were included (*N* = 332) and filled out a survey. The main variables studied in this research are shared decision‐making, behaviour (facilitating or limiting residents' alcohol use), care professionals personal alcohol use and their attitudes, person‐centred care and organisational culture. *t*‐tests, regression analyses and ANOVA analyses were conducted using SPSS.

**Results:**

Care professionals' attitudes towards residents' alcohol use are significantly associated with personal alcohol use; the level of SDM; and the level of enabling residents to drink alcohol. SDM is associated with person‐centred care (PCC) and significantly differs between somatic units and psychogeriatric units.

**Conclusion:**

SDM could be used in dilemmas regarding residents' alcohol use. Care professionals' personal alcohol use and attitudes towards residents' alcohol use could affect whether they discuss and facilitate this use. This may cause inconsistencies in care towards residents' alcohol use, which endangers PCC.

**Implications for Practice:**

SDM could support care professionals in dilemmas regarding residents' alcohol use. Care professionals should learn about the possible role of their personal alcohol use and attitudes regarding this topic. Finally, organizations should consider potential complicating factors when developing and implementing organizational policies regarding residents' alcohol use.

## Summary

**What does this research add to existing knowledge in gerontology?:**

- This empirical study adds knowledge to gerontology on care professionals' perspectives regarding alcohol use in older adults living in residential care facilities.
- This study also adds knowledge to gerontology on shared decision‐making regarding controversial habits of older adults living in residential care facilities, such as drinking alcohol.

**What are the implications of this new knowledge for nursing care for and with older adults?:**

- Shared decision‐making could support care professionals to involve residents in decisions regarding their alcohol use.
- Care professionals need to acknowledge the role of their personal alcohol use and attitudes regarding residents' alcohol use in the shared decision‐making process.

**How could the findings be used to influence practice, education, research, and policy?:**

- RCFs need to acknowledge the role of care professionals' personal characteristics when developing and implementing organisational policies regarding residents' alcohol use.
- Care professionals should be educated about the possible role of their personal alcohol use and of their attitudes on the care they provide on residents' alcohol use.

## 1 Introduction

Drinking alcohol has adverse (health) outcomes (Ortolá et al. 2024) and older adults are more sensitive to these adverse (health) outcomes (Kuerbis et al. 2014; Moore et al. 2007). Older adults who need to move to a residential care facility (RCF) due to physical or cognitive disabilities may wish to continue their alcohol use as they did at home. This use could vary from less than once per month to multiple glasses of alcohol per day (de Graaf et al. 2022). However, older adults who live in an RCF are dependent on their relatives or care professionals (Fazio et al. 2018), for example, to drink alcohol. This could jeopardise residents' autonomy regarding their wish to drink alcohol.

RCFs aim to provide person‐centred care (PCC) to older adults living in RCFs. PCC is a care paradigm that centres residents' values and preferences in daily care (Daly et al. 2018; McCormack and McCance 2021). The Dutch Care and Compulsion Act aims to enhance residents' autonomy and is in line with PCC. This act has been in effect since January 2020 and makes involuntary care (i.e., care against the will of a person, including restrictions) illegal, unless there is a risk of serious harm to the resident or to others (Ministerie van Volksgezondheid, Welzijn en sport 2021). The PCC paradigm and the Care and Compulsion Act restrict care professionals to limit residents' alcohol use when they wish to drink alcohol. However, care professionals tend to protect residents from the adverse outcomes of alcohol use and they might prefer to limit this use, as older adults are more sensitive to the adverse health outcomes of alcohol use (Kuerbis et al. 2014; Moore et al. 2007).

Care professionals have a crucial role in RCF residents' wish to drink alcohol (Bakhshi and While 2014; Johannessen, Tevik, Engedal, Gade Haanes, and Helvik 2021; Johannessen, Tevik, Engedal, and Helvik 2021). Their behaviour towards residents' alcohol use could be affected by their personal attitudes towards this use. The theory of planned behaviour suggests that personal beliefs influence attitudes, and, in turn, these attitudes affect care professionals' intentions and actions (Ajzen 1991), for example, regarding residents' wish to drink alcohol. Moreover, care professionals' beliefs and attitudes could also be affected by their personal alcohol use, which could have an impact on their professional performance (Albano et al. 2020; Verhoeven et al. 2024). Care professionals have a greater risk of hazardous alcohol use; for example, as a strategy to cope with distress and their highly demanding jobs (Albano et al. 2020; Mahmood et al. 2017; Searby et al. 2025; Searby et al. 2024). The provision of PCC, the Care and Compulsion Act and these abovementioned personal factors could create daily care dilemmas for care professionals when RCF residents wish to drink alcohol.

Shared decision‐making (SDM) is reported as a key element of PCC (McCormack and McCance 2021) and could support care professionals in dilemmas regarding residents' alcohol use. SDM consists of a continuous dialogue between residents, their relatives and care professionals (Cranley et al. 2020; van de Pol et al. 2016) and could be used for residents with both cognitive and physical disabilities. Although, the decision‐making capacity of RCF residents with primarily cognitive disabilities (e.g., dementia) gradually declines, which automatically increases the involvement of care professionals and relatives in the SDM process (Groen‐van de Ven et al. 2018).

Introducing SDM in RCFs regarding residents' alcohol use could enhance residents' involvement in decisions regarding their alcohol use and, in this way, enhance PCC. However, there are barriers to using SDM in RCFs (Cranley et al. 2020). First, care professionals may find it difficult to discuss residents' alcohol use (Johannessen, Tevik, Engedal, Gade Haanes, and Helvik 2021), especially in a society where alcohol continues to be socially accepted (Holdsworth et al. 2017). Objectively discussing this use could be affected by care professionals' attitudes towards alcohol and personal alcohol use (Bakhshi and While 2014; Johannessen, Tevik, Engedal, Gade Haanes, and Helvik 2021). In turn, this may affect the degree to which they facilitate or limit residents' alcohol use (Johannessen, Tevik, Engedal, Gade Haanes, and Helvik 2021). Second, the organisational culture may not sufficiently support SDM (Scholl et al. 2018). For example, a hierarchical culture is based on rules, structure and centralised decision‐making, which may decrease the level of SDM in a team of care professionals (van Beek and Gerritsen 2010).

This study aims to assess multiple factors that may affect the degree of SDM regarding residents' alcohol use and care professionals' behaviour (enabling or limiting) towards this use, resulting in the main research question: Do the attitudes and personal use of care professionals, the degree of PCC and the organisational culture have a relationship with the degree of SDM regarding residents' alcohol use and care professionals' behaviour (facilitating or limiting) towards this use?

## 2 Materials and Methods

### 2.1 Design

A quantitative cross‐sectional study was conducted, and explorative analyses were chosen to answer the research question. The Ethics Review Board from Tilburg University School of Social and Behavioural Sciences (Reference: TSB_RP531) granted approval. Additionally, approval was obtained from the scientific committees or executive boards of the participating organisations.

### 2.2 Study Setting and Sample

Care professionals providing 24/7 care to residents living in psychogeriatric or somatic units were included in this study. Psychogeriatric units provide 24/7 long‐term care to residents with mainly cognitive disabilities, such as dementia. Somatic units provide 24/7 long‐term care to residents with mainly physical disabilities. Previous research studied the residents' perspectives on this topic, and it was found that care professionals have an important role in limiting or facilitating residents' alcohol use (de Graaf et al. 2021, 2023). Therefore, we decided to include care professionals in this study, and we defined them as ‘nurse aides’, ‘vocational nurses’, ‘coordinating nurses’ and ‘registered nurses’. Participants with other functions related to the care of residents, such as ‘welfare employees’ or ‘hostesses’ were included in the category ‘other’.

Participants were recruited through convenience sampling from organisations that participate in a Dutch cooperative of six Academic Collaborative Centers Care for Older Adults (Samenwerkende Academische Netwerken Ouderenzorg, SANO), such as the Academic Collaborative Center Older Adults (ACC) (Luijkx et al. 2020).

Participants were recruited through team managers or coordinating nurses who invited care professionals personally or by e‐mail. We reached a sample of 272 participants through this process. Subsequently, a call was posted on multiple social media channels (LinkedIn, Facebook and Instagram) to reach the required sample size. This resulted in 60 more participants. All care professionals who were interested in participating received an information letter and an informed consent form (online or on a hard copy).

### 2.3 Data Collection

Data were collected between August 2023 and December 2023 using a survey of 70 questions. The data collection regarding alcohol use was part of a larger study (de Graaf et al. 2025). We added the questions that were used for this study in Appendix 1. Qualtrics was used for the online version, and the principal researcher (LG) transferred the hard copies to Qualtrics.

Data were collected on personal characteristics (e.g., age, gender, function and unit type in which the participant worked) and personal alcohol use. Personal alcohol use was assessed with the AUDIT‐C questionnaire (Bush et al. 1998), which consists of three questions to screen for hazardous alcohol use (Appendix 1). For this study, three groups are distinguished based on the cut‐off scores of the Dutch version from the Trimbos Instituut: those who never drink alcohol (AUDIT‐C sum score = 0), those who drink alcohol, but not at a hazardous level (AUDIT‐C sum score for men < 5 and for women < 4), defined as ‘regular’, and those who drink hazardous levels of alcohol (AUDIT‐C sum score for men ≥ 5 and for women ≥ 4). The AUDIT‐C is a screening instrument with good internal consistency (Cronbach's alpha = 0.94) (Meneses‐Gaya et al. 2010).

### 2.4 Independent Variables

Attitudes towards drinking alcohol in general and attitudes towards RCF residents drinking alcohol were measured with an attitude scale (ter Doest et al. 2009): seven items ranging from one to seven (1 = bad to 7 = good), resulting in a sum score (range 7–49). A higher score reflects a more positive attitude towards alcohol consumption (e.g., perceive drinking alcohol as more exciting and healthier). The scale was originally constructed for smoking tobacco and adjusted for this study by replacing the words ‘smoking tobacco’ with ‘drinking alcohol’. The original attitude scale was developed for adolescents based on the Theory of Planned Behaviour and has good internal consistency (Cronbach's alpha = 0.89) (ter Doest et al. 2009). Although adolescents were not our main study population, we choose to use this scale because it is short and easy to fill in by participants of all ages.

PCC was measured with the P‐CAT, which has good internal consistency (i.e., Cronbach's alpha = 0.84) and was tested in an Australian sample of long‐term care professionals (Edvardsson et al. 2010). The P‐CAT NL, the Dutch version, consists of 13 items that are rated on a five‐point Likert scale, ranging from one to five (1 = disagree completely to 5 = agree completely) (Trimbos Instituut 2010). This results in a sum score (range 13–65). A higher score means that care professionals report working in a more person‐centred approach.

Organisational culture was measured with the Dutch version of the Organizational Culture Assessment Instrument (OCAI), using an adapted version for better readability among care professionals who work in Dutch RCFs (van Beek and Gerritsen 2010). The questionnaire consists of six sets of four statements, with each statement reflecting a culture type. Each set is ranked in order from one to four (1 = least agree to 4 = most agree). This results in four sum scores (range 6–24) corresponding to four organisational culture types: A clan culture is a culture with strong cohesion, shared goals and values, participation and a sense of ‘us’. Adhocracy culture deals with rapid changes and adapts quickly to new opportunities. Market culture is a culture with focus on profitability and high result orientation. Finally, a hierarchy culture is based on rules, structure and centralised decision‐making (van Beek and Gerritsen 2010). The original version of the OCAI has sufficient internal consistency: the Cronbach's alpha coefficients of all scales were above 0.70 (Heritage et al. 2014; Kleijnen et al. 2009).

The unit type was assessed with one open‐ended question: ‘In what type of unit do you work?’ The answers were categorised into four types of units: ‘psychogeriatric’, ‘somatic’, ‘both psychogeriatric and somatic’ and ‘other’. The unit type defined as ‘other’ included, for example, units with a lower intensity of care provided to residents with less severe disabilities.

### 2.5 Dependent Variables

SDM regarding residents' alcohol use was both used as a dependent variable and an independent variable. It is used as an independent variable in the analyses of care professionals' behaviour (facilitating and limiting residents' alcohol use). SDM is assessed with the SDM‐Q‐Doc (Barr et al. 2014; Kriston et al. 2010): nine statements about SDM that participating care professionals rate on a six‐point Likert scale (0 = completely disagree to 5 = completely agree), resulting in a sum score (range 0–45). A higher score means a higher level of perceived SDM. The Dutch translation used in this study has good internal consistency (SDM‐Q‐Doc: Cronbach's alpha = 0.87) which was tested in a sample of general practitioners and medical specialists (Rodenburg‐Vandenbussche et al. 2015). Minor adaptations were made for this study by changing the word ‘patient’ into “resident” and adding that the decision applied to residents' alcohol use (e.g., ‘I made clear to the resident that a decision needs to be made about his/her alcohol use’).

Care professionals' behaviour (facilitating or limiting residents' alcohol use) was also used as a dependent variable. Based on the study of Kishore et al. (2011), the research team composed three questions to assess the degree of facilitating or limiting residents' alcohol use: ‘I help the resident to drink alcohol’; ‘I limit the resident in drinking alcohol’ and ‘I ask the resident about his or her history of alcohol use when they move to the RCF’. Participants rate these statements on a five‐point Likert scale (0 = never to 4 = always), resulting in a single score between zero and four for each statement. ‘Helping the resident to drink alcohol’ was defined in the broadest sense; for example, physically helping the resident to drink alcohol or providing alcohol when the resident wishes to drink alcohol and is physically unable to purchase alcohol.

### 2.6 Data Analysis

**FIGURE 1:**
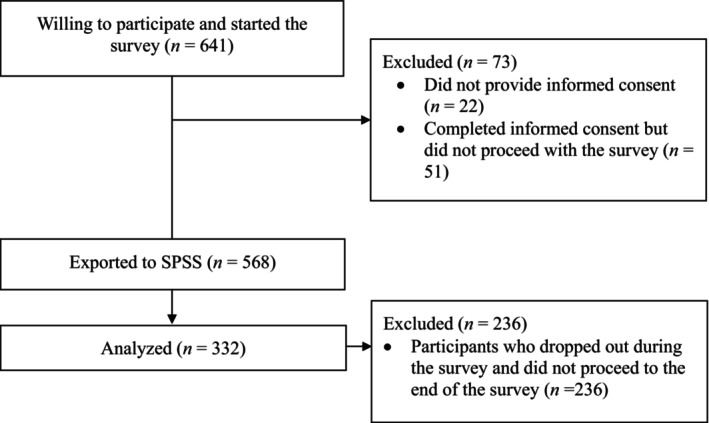
Flowchart of participants.

A power analysis was conducted to find an appropriate sample size before data collection started. A minimal sample size of 280 was needed to reach a power level of 0.95 with an alpha of 0.05 and an effect size of 0.15. Figure 1 shows a flowchart of the participants included in the final sample.

Data were analysed using SPSS version 28.0. Multiple linear regression analyses (‘enter’ method) were used to assess the associations between the independent and dependent variables. One‐way ANOVA was used to assess differences in three groups of personal alcohol use on SDM regarding residents' alcohol use and on care professionals' attitudes towards RCF residents drinking alcohol. Games‐Howell was used as post hoc analysis since the groups are unequal. Moreover, an independent samples *t*‐test was conducted to compare the degree of SDM between care professionals working in psychogeriatric units compared to those who work in somatic units. Those working in both psychogeriatric units and somatic units and those working in ‘other’ units were excluded from this *t*‐test. In the analyses regarding behaviour (enabling and limiting residents' use), weighted least square regressions were conducted because the assumption of homoscedasticity was violated. Moreover, in all analyses was controlled for potential confounding variables: age, function, level of education, unit type and personal alcohol use.

## 3 Results

A total of 332 care professionals participated in this study (age: *M* 44.64; SD 13.10; 91% female). Participating care professionals work in psychogeriatric units (47.9%), somatic units (25.3%), in both unit types (11.4%) and in other unit types (15.4%). From this sample, 16.0% of the care professionals do not drink alcohol, 61.7% regularly drink alcohol, and 22.3% report to drink alcohol at hazardous levels (Table 1). The descriptive statistics are presented in Table 2.

**TABLE 1:** Overview sample characteristics.

	*N*	%	*M* (SD)
Age			44.64 (13.10)
Gender (female)	302	91	—
Level of education			
Low (no education, elementary and vocational education)	45	13.6	—
Middle (secondary to high vocational education)	221	66.6	
High (high professional education and university)	65	19.6	
Unknown	1	0.3	
Type of unit			
Psychogeriatric	159	47.9	—
Somatic	84	25.3	
Psychogeriatric and somatic	38	11.4	
Other	51	15.4	
Function			—
Nurse aides	20	6	
Vocational nurses	99	29.8	
Coordinating nurses	52	15.7	
Registered Nurses	84	25.3	
Other	77	23.1	
Personal alcohol use			—
Group 1: non‐drinking	53	16.0	—
Group 2: not hazardous alcohol use	205	61.7	
Group 3: hazardous alcohol use	74	22.3	

**TABLE 2:** Descriptive statistics of the variables.

	*n*	*M* (range)	SD
Independent variables			
Attitude towards alcohol use in general	330	23.95 (7–49)	8.32
Attitude towards residents' alcohol use	329	25.74 (7–49)	9.10
Degree of PCC	330	46.53 (13–65)	6.52
Clan culture	291	14.45 (6–24)	6.45
Adhocracy culture	291	14.09 (6–24)	2.86
Market culture	291	13.86 (6–24)	5.30
Hierarchical culture	291	14.68 (6–24)	3.14
Dependent variables			
Degree of SDM	322	22.31 (0–45)	12.64
Facilitating residents to drink alcohol	329	1.83 (1–4)	0.81
Limiting residents in drinking alcohol	328	1.77 (1–4)	0.74

### 3.1 Care Professionals' Alcohol Use, Their Attitudes and SDM

A significant difference was found between the three levels of care professionals' alcohol use and their attitude towards RCF residents drinking alcohol (*F*[2, 326] = 5.176, *p* = 0.006). Post hoc analysis revealed that care professionals' attitudes towards residents' alcohol use significantly differ between the group of non‐drinkers (*M* = 22.57; SD = 1.13) compared to the group with hazardous alcohol use (*M* = 27.79; SD = 8.95) (*p* = 0.010). The groups did not significantly differ in the degree of SDM regarding residents' alcohol use (Table 3).

**TABLE 3:** Means, standard deviations and one‐way ANOVA of attitudes and SDM.

Measure	Non‐drinking		Not hazardous alcohol use		Hazardous alcohol use		*F*(2, 326)	*ⴄ* ^2^
	*M*	SD	*M*	SD	*M*	SD		
Attitudes towards RCF residents' alcohol use	22.57	10.13	25.85	8.67	27.79	8.95	5.176*	0.006
SDM towards residents' alcohol use	22.76	11.57	22.04	12.99	22.74	12.54	0.120	0.887

* *p* < 0.05.

### 3.2 Attitudes and SDM

Care professionals' attitude towards RCF residents drinking alcohol was significantly associated with the degree to which they provide SDM regarding residents' alcohol use (*β* = −0.115; *t*[293] = −1.978, *p* = 0.049) (Table 4). A more positive attitude towards residents drinking alcohol was associated with a lower degree of SDM towards residents' alcohol use. There was no significant association found between care professionals' attitudes towards drinking alcohol in general and the degree of SDM towards RCF residents drinking alcohol (Table 4).

**TABLE 4:** Multiple linear regression models for SDM.

	Estimate	SE	95% CI		*p*
			Lower bound	Upper bound	
Effect^a^, ^b^					
Attitude towards alcohol use in general	−0.072	0.096	−0.300	0.078	0.250
Attitude towards RCF residents' alcohol use	−0.115	0.081	−0.320	−0.001	0.049
Degree of PCC	0.284	0.109	0.345	0.774	< 0.001
Clan culture	0.016	0.119	−0.203	0.265	0.792
Adhocracy culture	0.083	0.277	−0.174	0.916	0.182
Market culture	0.007	0.145	−0.269	0.303	0.906
Hierarchical culture	0.026	0.245	−0.380	0.584	0.677

^a^ Dependent variable: degree of SDM.

^b^ Control variables: age, function, level of education, type of unit and personal alcohol use.

### 3.3 PCC, Organisational Culture and SDM

A significant association was found between the degree of PCC in general and the degree of SDM regarding residents' alcohol use (*β* = 0.284; *t*[295] = 5.129, *p* < 0.001): a higher degree of PCC was associated with a higher degree of SDM. The analysis of the organisational culture did not show any significant associations (Table 4).

### 3.4 Unit Type and SDM

The unit type was significantly associated with the degree of SDM regarding residents' alcohol use: care professionals in somatic units reported a higher degree of SDM (*M* = 25.17; SD = 11.25) than those in psychogeriatric units (*M* = 18.90; SD = 12.79) (*t*[234] = −3.895, *p* < 0.001).

### 3.5 Attitudes and Behaviours

A more positive attitude towards residents' alcohol use was significantly associated with more frequently facilitating residents to drink alcohol (*β* = 0.330; *t*[300] = 5.912, *p* < 0.001) (Table 4). No other significant associations were found with care professionals' behaviour (the degree of facilitating or limiting residents' use) (Table 5).

**TABLE 5:** Multiple linear regression models for behaviour^a^.

	Estimate	SE	95% CI		*p*
			Lower bound	Upper bound	
Effect^a^, ^b^, ^d^					
Degree of SDM	−0.039	0.004	−0.010	0.005	0.506
Attitude towards RCF residents' alcohol use	0.330	0.005	0.018	0.036	< 0.001
Effect^a^, ^c^, ^d^					
Degree of SDM	0.014	0.003	−0.006	0.008	0.812
Attitude towards RCF residents' alcohol use	−0.079	0.005	−0.016	0.003	0.177

^a^ Behaviour: facilitating or limiting residents' alcohol use.

^b^ Dependent variable: facilitating residents to drink alcohol.

^c^ Dependent variable: limiting residents in drinking alcohol.

^d^ Control variables: age, function, level of education, type of unit and personal alcohol use.

## 4 Discussion

SDM is reported as a key element of PCC (McCormack and McCance 2021) and could support care professionals in dilemmas regarding residents' alcohol use: considering residents' autonomy while protecting all residents and staff from adverse outcomes. Therefore, this study assessed multiple factors that could affect SDM regarding residents' alcohol use.

This study found that a substantial group of care professionals drink alcohol regularly (61.7%) and at hazardous levels (22.3%). These levels of personal alcohol use are also associated with care professionals' attitudes towards residents' alcohol use: a more positive attitude was associated with care professionals that drink alcohol at hazardous levels. Moreover, positive attitudes towards residents' alcohol use are related to a lower level of SDM and to more frequently facilitating residents to drink alcohol. Furthermore, this study found that a higher level of PCC is related to a higher level of SDM regarding residents' alcohol use; this was expected, as SDM is reported as a key element of PCC. Finally, care professionals working in somatic units report using more SDM regarding residents' alcohol use compared to those working in psychogeriatric units. This is in line with previous research on using SDM in general, but Groen‐van de Ven et al. (2018) emphasised that SDM is not yet fully incorporated in care provided to people with dementia. Further incorporating SDM within RCFs is in line with the Dutch Care and Compulsion Act, which encourages residents' autonomy and prohibits involuntary care in RCFs. Simultaneously, it may enhance PCC, though the SDM process needs to be adjusted to residents' cognitive capacities, which Groen‐van de Ven et al. (2018) also recommended.

This study supports results of previous studies that a substantial group of care professionals seems to drink alcohol regularly or at hazardous levels (Albano et al. 2020; Mahmood et al. 2017). The number of care professionals who may drink alcohol at hazardous levels (22.3%) is higher compared to the number of people that may drink at hazardous levels in the general Dutch population (12.7%) (Trimbos Instituut 2025). Moreover, a recent revision of Dutch guidelines for alcohol use disorders provided a stricter cut‐off score to screen for hazardous levels of alcohol use (for men ≥ 4 instead of ≥ 5 and for women ≥ 3 instead of ≥ 4) to increase screening sensitivity for hazardous alcohol use (Federatie Medisch Specialisten 2023; O'Connor et al. 2018). Following this revised guideline, the number of care professionals who screen positive for drinking alcohol at hazardous levels would be even larger (37% instead of 22.3%). The highly demanding nature of care professionals' jobs could further increase the risk of drinking alcohol at hazardous levels (Albano et al. 2020; Mahmood et al. 2017; Searby et al. 2025, 2024), which may impact the quality of care they provide to RCF residents (Albano et al. 2020; Schluter et al. 2012; Verhoeven et al. 2024). Therefore, this study indicates an urgency of addressing care professionals' personal alcohol use.

The study findings imply that the care professionals who view residents' alcohol use as more positive are more often facilitating residents drinking alcohol without using SDM. This is in line with the theory of planned behaviour, which suggests that personal beliefs influence attitudes, and, in turn, these attitudes affect care professionals' intentions and actions (Ajzen 1991). The role of these attitudes was also identified by Johannessen, Tevik, Engedal, Gade Haanes, and Helvik (2021), who reported a range of perceptions affecting the facilitation of residents' alcohol use: residents' alcohol use was perceived as a pleasure increasing their quality of life or as a potential threat to residents' health and well‐being. Alongside these attitudes, alcohol use continues to be socially accepted (Holdsworth et al. 2017), and care professionals may find it hard to discuss alcohol use with residents (Johannessen, Tevik, Engedal, Gade Haanes, and Helvik 2021). It appears that care professionals' attitudes are related to the level of SDM and to the level of facilitating residents' use. However, this may cause inconsistencies in care towards residents' alcohol use: when care professionals' attitudes determine whether they use SDM or whether they facilitate or limit residents' alcohol use, inconsistencies in care appear. These inconsistencies could endanger the provision of PCC as residents' values could become subordinate to care professionals' attitudes in care regarding residents' wish to drink alcohol.

### 4.1 Strengths and Limitations

A strength of this study is the focus on care professionals in RCFs as they are important in facilitating or limiting residents' alcohol use. This study contributes to the existing literature regarding factors that could affect SDM for residents' alcohol use; it affirms the relationship between PCC and SDM, and it reveals the role of care professionals' personal alcohol use and their attitudes on SDM and their behaviour towards residents.

However, there are also limitations that need to be considered. First, although the sample size was initially sufficient, the questionnaire for the organisational culture had the lowest response rate (87.7% of the participants filled out this part of the survey), despite the use of the adapted questionnaire by van Beek and Gerritsen (2010) to increase readability. Therefore, the results of this questionnaire have to be interpreted with caution. Second, data on the specific units where the participants worked were not collected to ensure the anonymity of the participants. Subsequently, clustering effects could not be measured, which further complicated the analyses of organisational culture. Third, care professionals' behaviour (facilitating or limiting residents' use) was assessed with only one question (‘I help the resident to drink alcohol’/‘I limit the resident to drink alcohol’). Therefore, data on care professionals' behaviour should be interpreted with caution.

### 4.2 Recommendations for Future Research

The results of this study may be repeated in a quantitative study with a greater sample size to affirm or contradict our results. Moreover, care professionals' behaviour could be assessed more extensively to understand which factors affect their behaviour regarding residents' wishes to drink alcohol. As described in the limitations, the current study assessed the behaviour with only one question, which is insufficient to draw firm conclusions on this part. Moreover, methodological issues arise when assessing actual behaviour through self‐report questionnaires. Qualitative studies, such as observational studies, could solve some of the methodological issues and can clarify the actual behaviour of care professionals. Therefore, future research should focus on alcohol use in residents living in RCFs with both qualitative and quantitative study designs. This may help to further understand why care professionals choose whether or not to use SDM or to facilitate or limit residents' alcohol use.

### 4.3 Implications for Policies and Practice

There are three implications for policies and practice. First, SDM could support care professionals in dilemmas regarding residents' alcohol use, but this study shows that multiple factors could complicate SDM regarding RCF residents' alcohol use. Second, care professionals should be educated about the possible role of their personal alcohol use and of their attitudes on the care they provide on residents' alcohol use. Third, RCFs need to acknowledge this role when developing and implementing organisational policies regarding residents' alcohol use.

## 5 Conclusion

SDM could be a helpful tool for care professionals to navigate dilemmas regarding residents' alcohol use. SDM exists within a context of national legislation and a society that still accepts drinking alcohol. Beyond this context, care professionals' personal alcohol use and attitudes may interfere with the use of SDM. Both the context and personal characteristics need to be considered when using SDM regarding RCF residents' alcohol use.

## Author Contributions

All authors contributed to the methodology and writing process: L.G. wrote the original drafts and T.R., M.J., S.B. and K.L. reviewed and edited the original drafts. L.G. was responsible for the data curation, data collection and project administration. L.G., T.R., M.J. and K.L. contributed to the conceptualisation of this manuscript. L.G., T.R. and S.B. conducted all formal analyses. This study is part of a larger study regarding alcohol and tobacco use in residential care facilities. The data collection was done simultaneously. The study regarding tobacco use was published earlier this year: de Graaf et al. (2025).

## Funding

This work was supported by Mijzo (Waalwijk, The Netherlands).

## Ethics Statement

The Ethics Review Board from Tilburg University School of Social and Behavioral Sciences (Reference: TSB_RP531) granted approval. Additionally, approval was obtained from the scientific committees or executive boards of the participating organisations.

## Consent

Participants received an information letter and an informed consent form, which was signed by them prior to participation (online or on a hard copy).

## Conflicts of Interest

The authors declare no conflicts of interest.

## Data Availability

The data that support the findings of this study are available on request from the corresponding author. The data are not publicly available due to privacy or ethical restrictions.

## Appendix 1 Survey

### Formal Caregivers

**Demographic features**

1. What is your age?
  _______________________________________________________________
2. What is your gender?
  ○ Male
  ○ Female
  ○ Other
  ○ Prefer not to say
3. What is your highest level of education?
  ○ Primary education
  ○ Secondary education (VMBO, HAVO/VWO year 1 to 3, MBO level 1)
  ○ Secondary vocational education (MBO level 2–4)
  ○ Higher vocational education (HBO)
  ○ Scientific/academic education (WO)
4. In which province do you work?
  __________________________________________________________________
5. In what type of unit do you work (psychogeriatric, somatic, etc.)?
  __________________________________________________________________
6. What is your job function?
  __________________________________________________________________

**Personal alcohol use**

7 How often do you have a drink containing alcohol? (Answer ‘never’? Continue to question 12).
  ○ Never (Continue to question 12)
  ○ Monthly or less
  ○ 2–4 times per month
  ○ 2–3 times per week
  ○ 4 or more times per week
8 How many units of alcohol do you drink on a typical day when you are drinking?
  ○ 1–2
  ○ 3–4
  ○ 5–6
  ○ 7–9
  ○ 10 or more
9 How often have you had 6 or more units on a single occasion in the last year?
  ○ Never
  ○ Less than monthly
  ○ Monthly
  ○ Weekly
  ○ Daily or almost daily

**Attitudes towards drinking alcohol**

People often have an opinion on certain topics, such as drinking alcohol. Fill in how you think about drinking alcohol.

10 Overall, I think drinking alcohol is:

**Table:**

	1	2	3	4	5	6	7	
Bad	○	○	○	○	○	○	○	Good
Dangerous	○	○	○	○	○	○	○	Not dangerous
Harmful	○	○	○	○	○	○	○	Not harmful
Unpleasant	○	○	○	○	○	○	○	Pleasant
Unhealthy	○	○	○	○	○	○	○	Healthy
Useless	○	○	○	○	○	○	○	Useful
Boring	○	○	○	○	○	○	○	Exciting

11 I think drinking alcohol in residents is:

**Table:**

	1	2	3	4	5	6	7	
Bad	○	○	○	○	○	○	○	Good
Dangerous	○	○	○	○	○	○	○	Not dangerous
Harmful	○	○	○	○	○	○	○	Not harmful
Unpleasant	○	○	○	○	○	○	○	Pleasant
Unhealthy	○	○	○	○	○	○	○	Healthy
Useless	○	○	○	○	○	○	○	Useful
Boring	○	○	○	○	○	○	○	Exciting

**Organisational culture**

Would you order the statements from low to high? The statement with which you agree the least regarding your unit gets a score of 1. The statement that you agree with most regarding your unit gets a score of 4.

12 This unit is:
  ______ a very personal place like belonging to a family
  ______ a very business‐like place with lots of risk‐taking
  ______ a very competitive place with high productivity
  ______ a very formal and structured place with lots of rules and policies

13 The unit supervisor is:
  ______ like a coach, a mentor or a parent figure
  ______ a risk‐taker, always trying new ways of doing things
  ______ a hard driver, very competitive and productive
  ______ a good organiser, an efficiency expert

14 The managing style at this unit is:
  ______ teamwork and group decision‐making
  ______ individual freedom to work in new ways
  ______ intense competition and getting the job done
  ______ job security, seniority system and predictability

15 My unit is held together by:
  ______ loyalty, trust and commitment
  ______ a focus on customer service
  ______ emphasising productivity, achieving goals and getting the job done
  ______ formal procedures, rules and policies

16 The work climate on my units:
  ______ promotes trust, openness and people development
  ______ emphasises trying new things and meeting new challenges
  ______ promotes competition and achievement of targets and objectives
  ______ pemphasises tradition, stability and efficiency

17 My unit defines success as:
  ______ teamwork and concern for people
  ______ being a leader in providing the best care
  ______ being number one when compared to other nursing homes
  ______ being efficient and dependable in providing services

**Person‐centred care**

18 Choose the degree to which you agree or disagree with these statements:

**Table:**

	Completely disagree	Disagree	Not agree or disagree	Agree	Completely agree
We often discuss how to give person‐centred care	○	○	○	○	○
We have formal team meetings to discuss residents' care	○	○	○	○	○
The life history of the residents is formally used in the care plans we use	○	○	○	○	○
The quality of the interaction between staff and residents is more important than getting the tasks done	○	○	○	○	○
We are free to alter work routines based on residents’ preferences	○	○	○	○	○
Residents are offered the opportunity to be involved in individualised everyday activities	○	○	○	○	○
Assessment of residents’ needs is undertaken on a daily basis.	○	○	○	○	○
I simply do not have the time to provide person‐centred care	○	○	○	○	○
The environment feels chaotic	○	○	○	○	○
We have to get the work done before we can worry about a homelike environment	○	○	○	○	○
This organisation prevents me from providing person‐centred care	○	○	○	○	○
It is hard for residents in this facility to find their way round	○	○	○	○	○
Residents are able to access outside space as they wish	○	○	○	○	○

**Shared decision‐making regarding (daily) care of the resident**

19 Think about daily work situations where decisions had to be made regarding residents’ alcohol use.

Answer the questions below regarding residents’ alcohol use. Fill in the degree to which you agree or disagree with each statement. Fill in one answer per statement:

**Table:**

	Completely disagree	Strongly disagree	Somewhat disagree	Somewhat agree	Strongly agree	Completely agree
I made clear to the resident that a decision needs to be made	○	○	○	○	○	○
I wanted to know exactly from the resident how he/she wants to be involved in making the decision	○	○	○	○	○	○
I told my patient that there are different options regarding drinking alcohol	○	○	○	○	○	○
I precisely explained the advantages and disadvantages of the treatment options to the resident	○	○	○	○	○	○
I helped the resident understand all the information	○	○	○	○	○	○
I asked the resident which option he/she prefers	○	○	○	○	○	○
The resident and I thoroughly weighed the different options	○	○	○	○	○	○
The resident and I selected an option together	○	○	○	○	○	○
The resident and I reached an agreement on how to proceed	○	○	○	○	○	○

**Behaviour of care professionals**

20 Fill in the degree to which these statements are true about your work in the past week. Fill in one answer per statement.

Answer these statements about residents’ alcohol use:

**Table:**

	Never	Sometimes	Often	Always
I help the resident to drink alcohol	○	○	○	○
I limit the resident in drinking alcohol	○	○	○	○
I ask the resident about his or her history of alcohol use when they move to the RCF	○	○	○	○
